# The use of telehealth in attention-deficit/hyperactivity disorder: a survey of parents and caregivers

**DOI:** 10.1007/s00787-024-02466-y

**Published:** 2024-05-16

**Authors:** Emer Galvin, Blánaid Gavin, Ken Kilbride, Shane Desselle, Fiona McNicholas, Shane Cullinan, John Hayden

**Affiliations:** 1 School of Pharmacy and Biomolecular Sciences, Royal College of Surgeons in Ireland, Dublin, Ireland; 2 School of Medicine, University College Dublin, Dublin, Ireland; 3 ADHD Ireland, Dublin, Ireland; 4 Touro University California, Vallejo, CA USA; 5 Children’s Health Ireland, Crumlin, Dublin, Ireland; 6 Lucena Child and Adolescent Mental Health Service (CAMHS), Rathgar, Dublin, Ireland

**Keywords:** ADHD, COVID-19, Telehealth, Remote assessment, Parents

## Abstract

The use of telehealth became widespread during the COVID-19 pandemic, including in child and adolescent attention-deficit/hyperactivity disorder (ADHD) services. Telehealth is defined as live, synchronous phone and video appointments between a healthcare provider and a parent and/or child with ADHD. There is a dearth of research on the use of telehealth within this population. The aim of this study was to examine parents’ and caregivers’ perceptions of telehealth for children and adolescents with ADHD. A cross-sectional survey design was employed. Recruitment of parents and caregivers of children and adolescents with ADHD was conducted online. The survey asked participants about their views of telehealth, previous experience, and willingness to use telehealth. Quantitative data were analysed using STATA. Qualitative data were analysed using content analysis. One hundred and twelve respondents participated in the survey. Participants were mostly female (n = 97, 86.6%) and aged between 45 and 54 (n = 64, 57.1%). Of the 61 (54.5%) participants with experience of telehealth, the majority reported that that they were at least satisfied with telehealth visits (n = 36, 59%), whilst approximately half rated their quality more poorly than in-person visits (n = 31, 50.8%). The majority of respondents (n = 91, 81.3%) reported that they would be willing to use telehealth for their child’s future appointments. Most common reasons selected for wanting to use telehealth included saving time, improvements to the family routine, and reducing costs. Reasons selected for not wanting to use telehealth included not being able to receive hands-on care, belief that the quality of care is poorer than in-person consultations, and distraction of the child during telehealth visits. The study demonstrates that parents recognise deficits and benefits of telehealth, suggesting a need to build their trust and confidence in remote ADHD care.

**Supplementary Information:**

The online version contains supplementary material available at 10.1007/s00787-024-02466-y.

## Introduction

Attention-deficit/hyperactivity disorder (ADHD) is a neurodevelopmental condition characterised by developmentally-inappropriate levels of inattention, hyperactivity, and impulsivity [1]. It is the most common neurodevelopmental condition presented to child and adolescent mental health services (CAMHS) [2]. It is acknowledged that ADHD may be a condition that is suitable for telehealth, as it is highly prevalent and there are well-established guidelines for the assessment and management of ADHD [3, 4]. In the present study, telehealth is defined as live, synchronous phone and video appointments between a healthcare provider and a parent and/or child with ADHD. This includes the use of phone and video appointments for assessments, psychological interventions, medication reviews, and other appointments that involve the aforementioned individuals.

Children and adolescents with neurodevelopmental conditions and their families often face barriers to accessing treatment [5], similar to other enduring health conditions. Structural barriers include financial costs, taking time off work, lack of treatment availability, and travelling long distances to specialised clinics [5, 6]. Furthermore, psychological barriers that impede engagement with paediatric ADHD services include caregiver distress and strain [7], stigma of ADHD and treatment [8], and low caregiver self efficacy to access and implement treatment [9]. For children and adolescents with neurodevelopmental conditions, travelling to appointments and attending new medical environments can exacerbate behaviours and be a source of psychological stress [5, 10, 11]. Telehealth has the opportunity to address structural and psychological barriers to treatment by increasing access, but also reducing caregiver stress, increasing engagement with services, and improving quality of life [5, 6]. Prior to the pandemic, telehealth had not been used extensively for the provision of care for children or adults with ADHD. A review by Spencer and colleagues [12] identified only eleven studies investigating the use of telehealth in ADHD, eight of which were from one trial. Notably, they found no studies that examined telehealth as a replacement for in-person care [12].

As with all mental health services, the COVID-19 pandemic transformed ADHD services, with clinicians required to quickly adapt their practice to reduce in-person contact and stop the spread of the virus. A prominent change was the move from in-person appointments to video and phone appointments to conduct assessments and consultations. The pandemic disrupted care for existing patients and reduced access for new patients [4]. The associated lockdowns exacerbated the problems experienced by people with ADHD [13, 14], and their families, including increases in caregiver distress and burden [15]. Whilst in-person ADHD services have resumed, telehealth remains as a method for the provision of care.

Research has begun to investigate this recent increase in the use of telehealth for children and adolescents with ADHD and other neurodevelopmental conditions. In relation to effectiveness, a recent study has demonstrated the reliability and feasibility of telehealth for the assessment of ADHD among a sample of Japanese children [17]. In addition, a qualitative service evaluation of caregiver perspectives of remote ADHD assessments in a United Kingdom (UK) CAMHS highlighted comfort, convenience, and flexibility of remote assessments as their positives [18]. A study exploring the impact of COVID-19 restrictions on children with ADHD in Australia found that parents rated telehealth as being the same (42%) or poorer (48%) quality compared to in-person appointments [16]. Finally, challenges of telehealth for children and adolescents with neurodevelopmental conditions have been reported by caregivers, including concerns about the need for parental involvement in telehealth [17], concerns that providers could be missing behavioural cues [18], and issues with technical difficulties [5].

Ros-DeMarize and colleagues [19] state that given the variety of factors that impact the remote delivery of ADHD services, it is vital to understand the child, parent, familial, and community factors that impact engagement. Research is particularly limited on parent and caregiver perspectives of telehealth within neurodevelopmental conditions [20], despite being recognised as important for informing future telehealth services [4, 21].

The overall aim of this study was to explore parents’ and caregivers’ perspectives of the use of telehealth for children and adolescents with ADHD. The specific objectives were:

1. To evaluate parents’ and caregivers’ opinions of telehealth
2. To measure parents’ and caregivers’ willingness to use telehealth
3. To explore reasons for wanting, and not wanting, to use telehealth

## Methods

### Survey design

This study employed a cross-sectional survey design. An online, study-specific survey was designed using the REDCap^©^ platform [22, 23].

### Sampling and recruitment

The sample comprised parents and caregivers of children and adolescents with ADHD. Recruitment was facilitated by our Patient and Public Involvement (PPI) partner, ADHD Ireland. ADHD Ireland is an organisation that provides information, resources, and networking opportunities to people with ADHD, parents and caregivers of children with ADHD, and the professionals who serve them. ADHD Ireland is a registered charity and receives funding from the Irish government. The website is managed by an employee of ADHD Ireland. The survey was advertised on the ADHD Ireland website and the research call was distributed to approximately 3500 parents and caregivers of children and adolescents with ADHD via ADHD Ireland’s email newsletter (See Appendix 1 for the research call).

#### Eligibility criteria

Participants were eligible for inclusion if they were a parent or a caregiver to a child or adolescent with ADHD. They must have been aged over 18. Participants were not eligible if they were unable to give informed consent and if they did not reside in Ireland. Participants must have been able to comprehend English.

#### Sample size calculations

Sample size calculations were conducted in STATA 17 [24] using the *svysampsi* command for surveys with a dichotomous outcome variable, with a population size of 3500, a proportion of 50% assumed to have the expected outcome, an error rate of 10%, and a 95% confidence interval [25]. This gave an estimated sample size of 95 participants. A proportion of 50% was used as an expected proportion of participants willing to use telehealth. Recent studies of parents of paediatric patients have reported high proportions of willingness to use telehealth beyond the pandemic (87% [5], 81.3% [26]). However, the samples in these studies had previous experience with telehealth, so a more conservative estimate of 50% was chosen. A 10% margin of error was used, as this has been considered appropriate in the literature [27].

### Survey development

A 32-item study-specific survey was created using the REDCap^©^ platform (See Appendix 2). REDCap^©^ is a cloud-based data collection platform that guarantees security for stored data. To achieve the study aims, a scoping search of the literature was conducted to identify relevant topics for inclusion. No survey measures to examine perceptions of telehealth for ADHD were identified. The lead researcher (EG) led the development of the survey. It was informed by relevant surveys of telehealth outside of ADHD. Our PPI partner, ADHD Ireland, was consulted and involved in the co-design of the survey. An important consideration was that many parents of children with ADHD have ADHD themselves, so the survey should be easy to follow and efficient to complete.

Following a rigorous development protocol, the survey underwent expert review by two child and adolescent psychiatrists, who suggested additional items for inclusion and changes to the wording and structure of the survey. Our PPI partner also reviewed the survey for readability, relevance, and easiness-of-completion. The final survey was reviewed by members of the research team (EG, JH, SC) for content, clarity, and length. These researchers have expertise in survey design and analysis, research with parent and paediatric populations, and mixed methods research. Further amendments were incorporated and the survey was then tested with lay members of the public for functionality and clarity. Finally, the survey was piloted with two parents to ensure readability and face validity. These parents comprised a male and female both aged 45–54, both with two children with ADHD, residing in two distinct areas in Ireland. These pilot responses were not included in the final analysis.

The final survey included a combination of open- and closed- ended questions. In this study, “telehealth” is defined as live, synchronous phone and video appointments between a healthcare provider and a parent and/or child with ADHD. The quantitative data required categorical responses, responses on Likert scales, and rating and ranking of multiple-choice options. The survey included questions on willingness to use telehealth, potential uses for telehealth, and previous experience of telehealth. Participants were also asked about demographic information and about access to technology. The open-ended questions elicited responses about (1) additional reasons for wanting to use telehealth, (2) additional reasons for not wanting to use telehealth, and (3) additional comments about telehealth.

### Data collection

Data were collected between July and August 2023. Individuals were emailed a link to the survey via the ADHD Ireland email newsletter. On opening the link, participants were presented a participant information leaflet. They were asked to complete an online consent form before commencing the survey. The survey took participants approximately 10 to 15 min to complete. On completion of the survey, participants were presented with a thank you note and with the lead researcher’s contact details. Data were collected until the estimated minimum sample size was reached. A reminder email was sent two weeks following the initial email.

### Data analysis

Quantitative data were analysed using STATA 17. Descriptive statistics were used to describe survey responses and were represented as frequencies (n) and percentages (%). Pearson’s Chi-squared tests and Fisher’s exact tests were used to examine any differences between categorical variables and willingness to use telehealth. Statistical comparisons were two-tailed and statistical significance was set at p < 0.05. Qualitative data from the open-ended questions were analysed using content analysis [28]. The aim of this analysis was to identify additional reasons participants cited for wanting, and not wanting, to use telehealth. The lead researcher (EG) conducted a conceptual content analysis, whereby the existence and frequency of concepts were determined [28]. The data were coded at the level of the phrase, and data from the three open ended questions were analysed simultaneously. The data were grouped into categories to address the open-ended questions. Finally, the frequency of each concept was recorded and the final concepts were presented narratively alongside the quantitative data for reasons for, and against, using telehealth.

### Patient and public involvement

As described above, a representative from ADHD Ireland was involved in the design of the study and the survey instrument. Survey piloting and data collection were facilitated by ADHD Ireland. The representative was an individual in a leadership position within ADHD Ireland. They met with the research team via online meetings and communicated with the lead researcher (EG) via email. The representative was primarily consulted in the design and review of the survey instrument and information materials. The representative also acted as a gatekeeper for pilot participants and was consulted in the reporting of the findings. The representative was not compensated for their time but was included in the study authorship (KK). We aim to disseminate the findings to relevant knowledge users with ADHD Ireland.

### Ethics and informed consent

This study received ethical approval from the Royal College of Surgeons in Ireland Research Ethics Committee (Reference number: 212627453). Participation was voluntary, anonymous, and confidential. Participants were required to read an information sheet and indicate their consent via an opt-in tick box at the start of the survey. Participants were made aware that they could withdraw from the survey at any point, although it would not be possible to withdraw after completion of the survey, due to the anonymous nature of the survey responses. The study is reported in accordance with the Checklist for Reporting Results of Internet E-Surveys (CHERRIES) [29] (See Appendix 3).

## Results

The survey was distributed to approximately 3500 parents and caregivers of children and adolescents with ADHD via ADHD Ireland. In total, 121 participants consented to participate in the survey. Nine participants completed less than 50% of the survey, so were excluded from the analysis. A total of 112 participants were included in the final analysis (response rate: 3.2%).

### Demographic characteristics

The majority of participants were female (n = 97, 86.6%), and aged between 45 and 54 (n = 64, 57.1%). Participants were primarily parents (n = 109, 97.3%). Most participants resided in county Dublin (n = 42, 37.5%) and in the province of Leinster (n = 75, 67%). Twenty-two participants had a diagnosis of ADHD themselves (19.6%). The full demographic characteristics of the sample can be seen in Table 1.

**Table 1:** Participant demographic information (N = 112)

Characteristic		n (%)
Age		
25–34		3 (2.7%)
35–44		28 (25%)
45–54		64 (57.1%)
55–64		17 (15.2%)
Gender		
Male		12 (10.7%)
Female		97 (86.6%)
Missing		3 (2.7%)
Role		
Parent		109 (97.3%)
Guardian		1 (0.9%)
Missing		2 (1.8%)
Province		
Leinster		75 (67%)
Munster		24 (21.4%)
Connacht		10 (8.9%)
Ulster		1 (0.9%)
Missing		2 (1.8%)
Parent ADHD diagnosis		
Yes		22 (19.6%)
No		65 (58%)
No, but I think I may have ADHD		25 (22.3%)

The majority of participants reported having one child with ADHD (n = 86, 76.8%). Of the 145 children with ADHD, 95 were male (65.5%). The age range of children were 4 to 30 with a mean age of 14.8 years (SD = 5 years). The demographic information of the children are reported in Table 2.

**Table 2:** Child demographic information

Characteristic	n (%)
Number of children with ADHD (N = 112)	
1	86 (76.8%)
2	19 (17%)
3	3 (2.7%)
4 or more	3 (2.7%)
Missing	1 (0.8%)
Gender of child (n = 145)	
Male	95 (65.5%)
Female	48 (33.1%)
Other	1 (0.7%)
Missing	1 (0.7%)
Age of child (n = 145)	
4–10	30 (20.7%)
11–15	55 (37.9%)
16–19	36 (24.8%)
20–30	24 (16.6%)

### Technology access and use

All participants reported having access to at least one technological device. Smartphones were the most commonly owned device (n = 111, 99.1%), followed by laptop computers (n = 104, 92.9%), tablets (n = 81, 72.3%), and gaming consoles (n = 80, 71.4%). The majority of participants (n = 108, 96.4%) accessed the internet at least several times a day. Most participants used video-based platforms (e.g. Zoom) to communicate with others at least once a week (n = 83, 74.1%).

### Previous experience of telehealth

Sixty-two (55.4%) participants reported having used telehealth for general healthcare appointments. Only 17 (15.2%) participants reported using telehealth prior to the COVID-19 pandemic. Sixty-one (54.5%) participants reported having used telehealth for ADHD appointments. Of these, the majority reported using telehealth for ADHD appointments more than once (n = 55, 90.2%). Most participants reported using mainly video appointments (n = 29, 47.5%), followed by phone appointments (n = 14, 23%), and an equal mix of both phone and video appointments (n = 11, 18%). Within this subsample, telehealth was most often used for general appointments (n = 35, 57.4%), followed by medication reviews (n = 32, 52.5%), and therapeutic appointments (n = 27, 44.3%).

### Satisfaction with telehealth

The subsample of respondents who had previous experience with telehealth for ADHD appointments (n = 61) were asked to rate their satisfaction with telehealth across six questions. The survey items regarding satisfaction are presented in Appendix 4. Most participants reported that they found telehealth easy, or very easy, to use (n = 43, 70.5%) and that they were either somewhat, or very, satisfied with the privacy/security of telehealth (n = 43, 70.5%). The majority of participants (n = 38, 62.3%) reported that they were at least comfortable communicating with their healthcare professional via telehealth. Much less (n = 21, 34.4%) participants reported that their child was at least comfortable communicating with their healthcare professional via telehealth. Roughly half of the participants believed that the quality of telehealth was poorer than in-person visits (n = 31, 50.8%). Finally, the majority of participants were either somewhat satisfied, or very satisfied, with telehealth (n = 36, 59%).

### Future use of telehealth

**Fig. 1:**
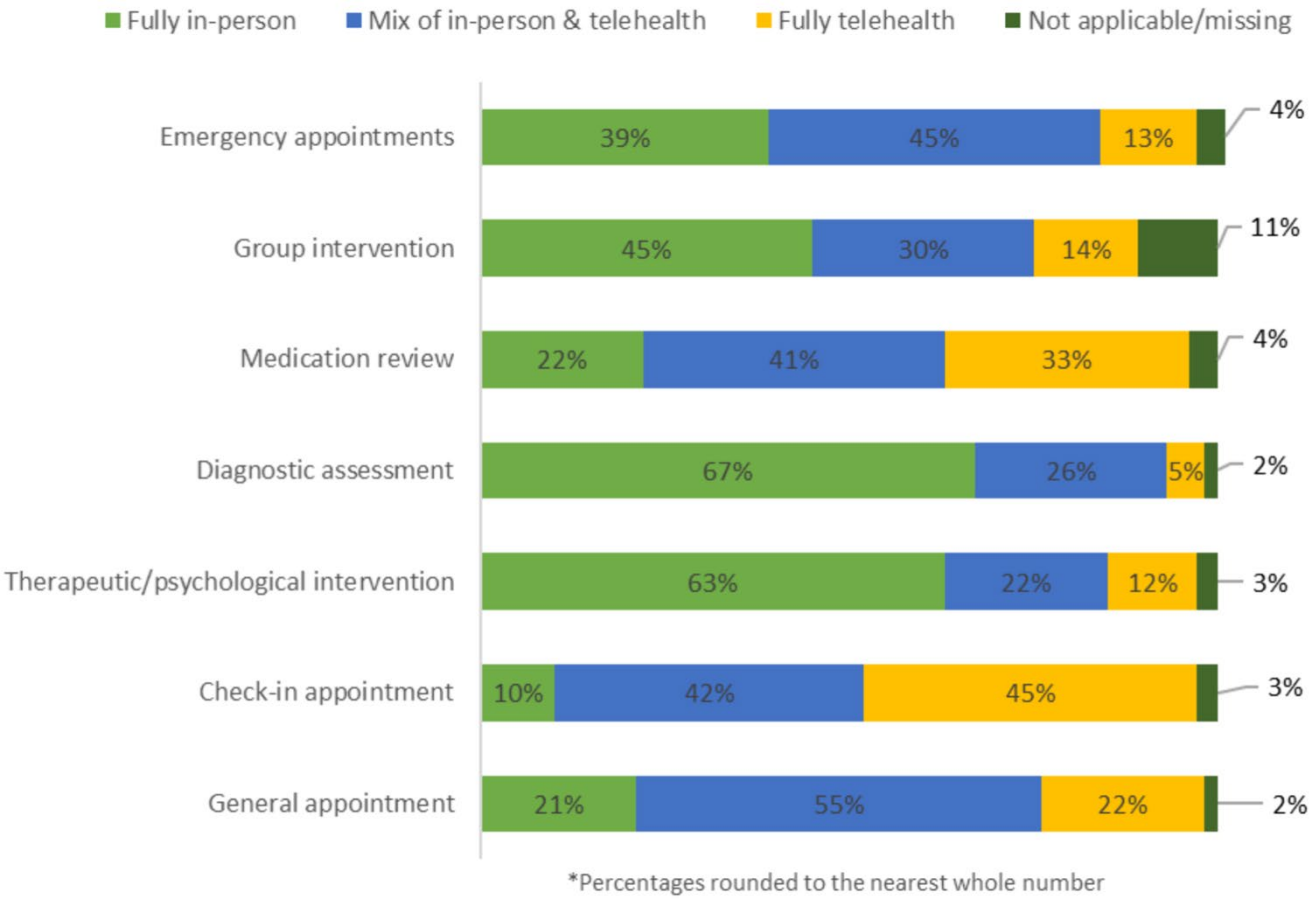
Use of telehealth for future ADHD appointments (N = 112)

Ninety-one (81.3%) participants reported being willing to use telehealth for future ADHD appointments. Participants were asked to indicate what proportion of various appointment types they would be willing to use telehealth for (fully in-person, mixed of in-person and telehealth, fully telehealth). Participants reported a preference for using telehealth exclusively for check-in appointments (n = 50, 44.6%), followed by medication reviews (n = 37, 33%), and general appointments (n = 25, 22.3%). Participants reported a preference for using in-person appointments exclusively for diagnostic assessments (n = 75, 67%), therapeutic or psychological interventions (n = 71, 63.4%), and group interventions (n = 50, 44.6%) (See Fig. 1).

The associations between several variables and participants’ willingness to use telehealth (yes vs no) were examined using Chi-squared tests of independence and Fisher’s exact tests. There was no association between age group, gender, having used telehealth previously for general appointments, or having used telehealth previously for ADHD appointment and willingness to use telehealth. There was a significant association between using telehealth before the pandemic (yes vs no) and willingness to use telehealth (Fisher’s Exact Test, 2-tailed p = 0.04). Of note, all 17 participants who reported using telehealth before the pandemic reported that they would be willing to use telehealth in the future.

Within the subsample who had used telehealth for ADHD appointments previously, there was a significant association between satisfaction with telehealth (satisfied/neutral vs unsatisfied) and willingness to use telehealth (Fisher’s exact test 2-tailed, p < 0.001). There was also a significant association between perception of quality of care compared to in-person appointments (worse vs equal/better) and willingness to use telehealth (yes vs no) (Fisher’s exact test 2-tailed, p = 0.002). Of note, 19 participants who rated the quality of telehealth worse than in-person visits reported being willing to use telehealth going forward. Finally, there was no association between the amount of times telehealth was used (five times or less vs six times or more) and willingness to use telehealth (χ2 = 0, p = 0.99). The results of these tests are presented in Appendix 4.

### Reasons for wanting to use telehealth

**Fig. 2:**
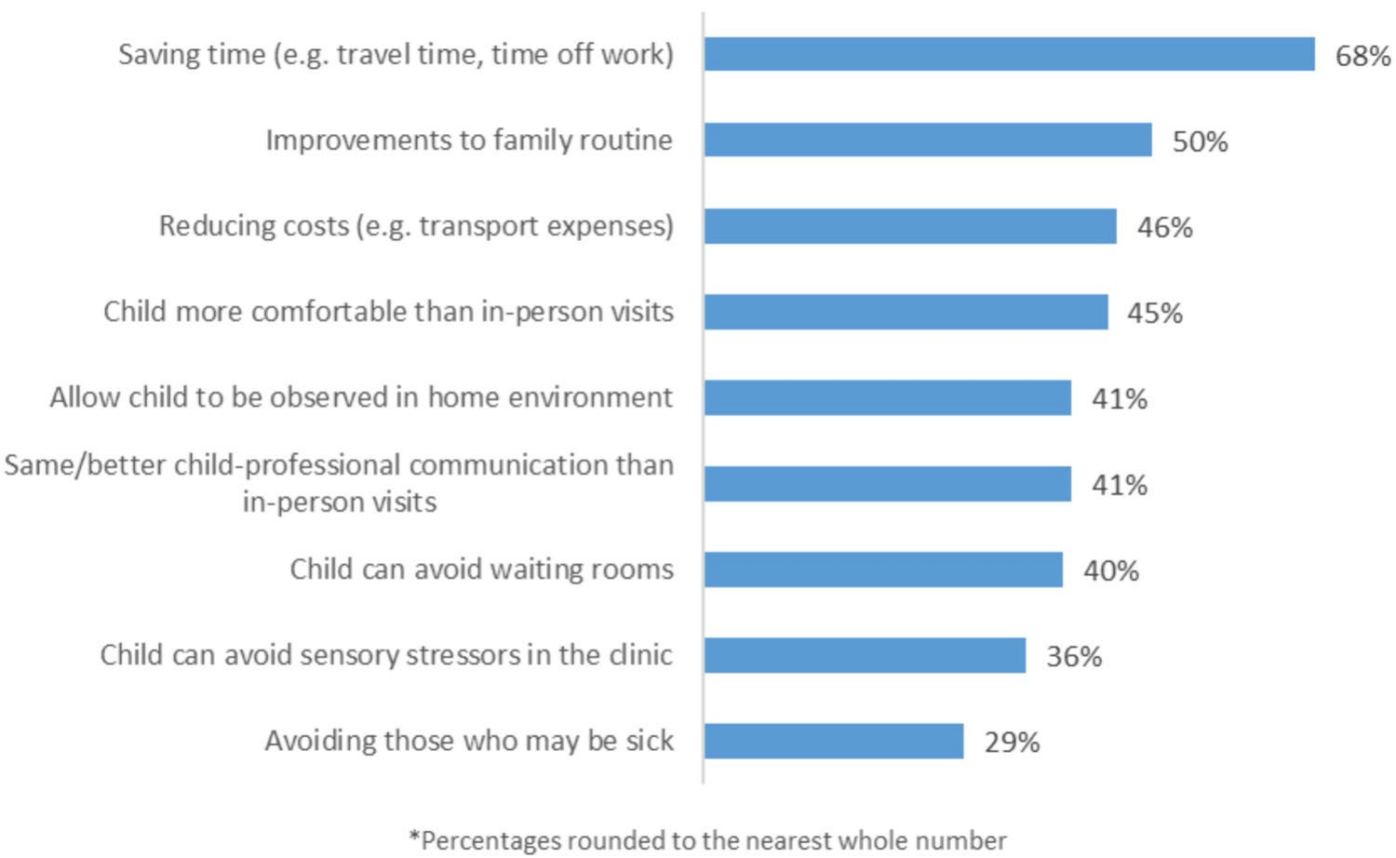
Reasons selected for wanting to use telehealth (N = 112)

Participants were asked to rank a list of reasons for wanting to use telehealth, from most important to least important. They could rank up to five reasons and had the option to skip the question. The most frequently selected reasons included saving time (n = 76, 67.9%), improvements to family routine (n = 56, 50%), and reducing costs (n = 51, 45.5%). The full list of reasons, and frequencies, are presented in Fig. 2.

Participants were also asked to provide additional reasons why they would be willing to use telehealth in the future. The most frequent comments related to getting quicker access to services and the presence of long waiting lists for in-person services (25 comments). Participants noted that they would be willing to use telehealth for check-in appointments and medication reviews, and as a complement to in-person care (16 comments). Participants also mentioned that telehealth was a suitable option for their child who was not comfortable with in-person appointments (eight comments). Further comments expanded on the reasons presented in the survey, such as the convenience aspects of saving time, reducing travel, not having to get childcare, and not having to take time off work and school (22 comments). Improvements to the family routine were discussed, including avoiding stress and disruption of attending in-person appointments for both parent and child (12 comments). Specifically, for parents with neurodivergencies, telehealth eased some of the burdens of attending in-person appointments. Participants also highlighted the utility of telehealth in emergency situations and when their child was ill (seven comments). Finally, a small number of participants (three comments) highlighted that telehealth is more suited to the family situation, allowing both parents to attend and facilitating male partners to be more involved in their child’s care. Illustrative quotes from the free-text comments are presented in Appendix 5.

### Reasons for not wanting to use telehealth

**Fig. 3:**
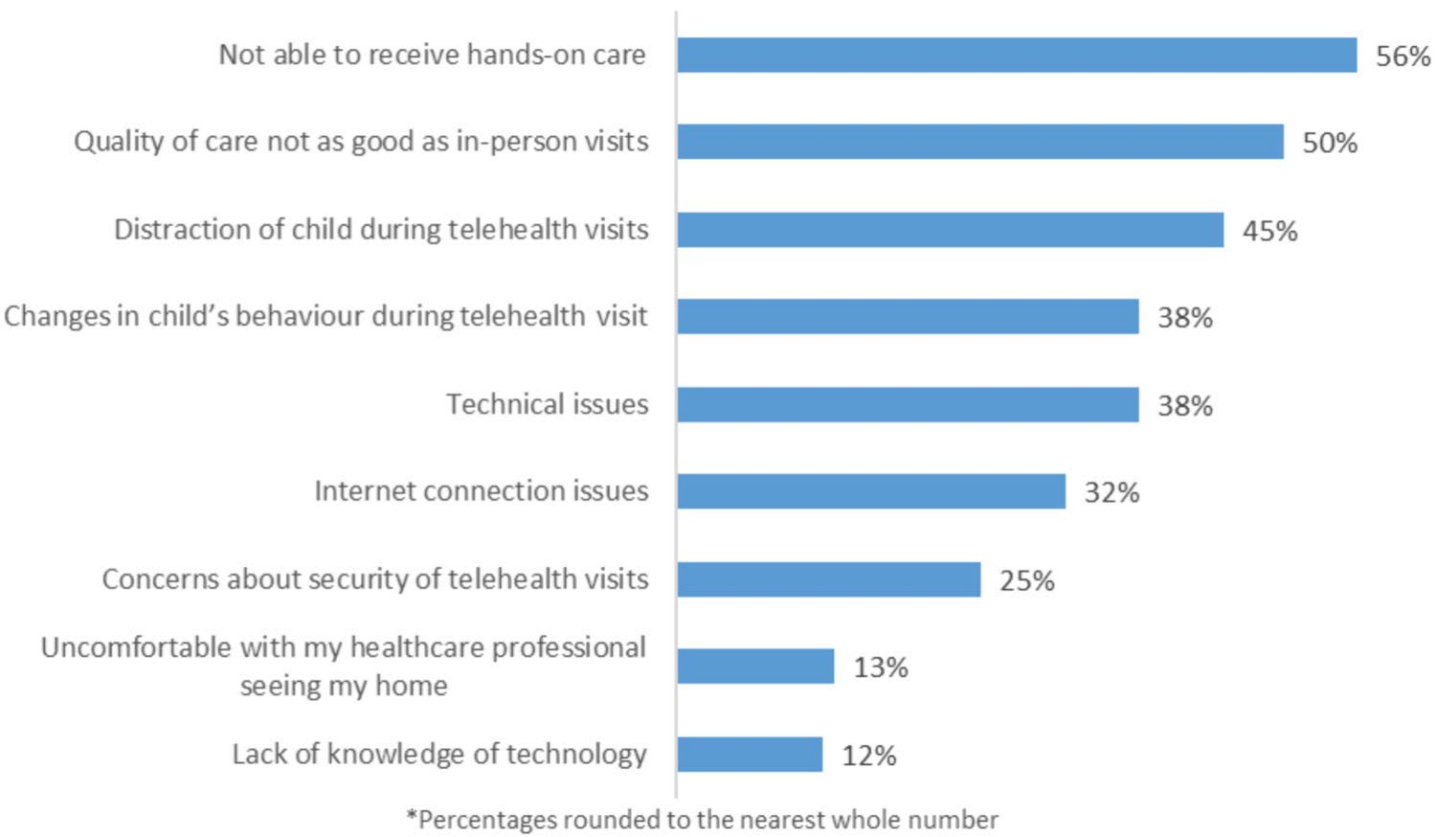
Reasons selected for not wanting to use telehealth (N = 112)

Participants were also asked to rank a list of reasons for not wanting to use telehealth. The most frequently selected reasons included not being able to receive hands-on care (n = 63, 56.3%), believing that the quality of care of telehealth is not as good as in-person visits (n = 56, 50%), and distraction of the child during telehealth visits (n = 50, 44.6%). The full list of reasons, and frequencies, are presented in Fig. 3.

Participants were also asked to provide any additional reasons why they would not be willing to use telehealth in the future. The main reason was difficulty of the child engaging in phone and video calls, including getting distracted on calls with the healthcare provider (25 comments). Preference for, and necessity of, in-person appointments were also discussed (15 comments). The perceived impaired quality of interactions (10 comments) and concerns about the accuracy of diagnosis and assessment over telehealth (14 comments) were described. Finally, the inability of the clinician to perform physical examinations (e.g. blood pressure) were mentioned by participants (five comments). Illustrative quotes from the free-text comments are presented in Appendix 5.

## Discussion

### Main findings

The aim of this study was to examine parents’ and caregivers’ perceptions of telehealth for children and adolescents with ADHD. Just over half of participants had experience using telehealth for ADHD appointments. Most of these participants reported that telehealth was easy to use and that they were satisfied with telehealth. Approximately half of these participants rated the quality of telehealth as poorer than in-person visits. Satisfaction and positive perceptions of quality of care were significantly associated with willingness to use telehealth. Overall, the vast majority of participants reported willingness to use telehealth. However, in-person care was valued for certain appointments, namely diagnostic appointments and therapeutic interventions. The free-text comments highlighted beliefs that telehealth was inferior to in-person care in terms of the quality of assessment and the interaction between the child and clinician. Some participants reported willingness to exclusively use telehealth for certain appointments, namely check-in appointments and medication reviews. The convenience aspects of telehealth were appreciated here, as was the timelier availability of telehealth appointments. An interesting finding was that there was a significant association between having used telehealth before the pandemic and willingness to use telehealth going forward, whereby all participants who reported pre-pandemic use were willing to continue to use telehealth.

### Comparison with previous research

Our study addresses the dearth of research of parent and caregiver perceptions of telehealth for ADHD. The findings can be situated within the broader research area of parental perceptions of telehealth for children and adolescents. As with other studies of parental perceptions of telehealth [30, 31], the majority of respondents were female (over 80%), which may represent the disproportionate care responsibilities of female caregivers. As highlighted by a small number of female participants, telehealth allowed for the male parents to be more involved in their child’s care. The majority of parents with experience of telehealth for ADHD appointments reported satisfaction, which is consistent with previous studies of parental perspectives [5, 26, 30–32]. In other studies exploring views of parents of telehealth [5, 26], the most prevalent barriers pertained to technical difficulties. This contrasts with our findings that telehealth was generally easy to use and the relatively low number of people who cited lack of technical knowledge as a reason for not wanting to use telehealth. From the questions on technology use, it appears our sample was technologically literate, which may explain this difference. However, technical issues were rated by approximately a third of participants as a reason for not wanting to use telehealth, suggesting issues may lie with the platforms themselves rather than the technological competency of the parents.

Other studies have also reported high levels of willingness to use telehealth among parents of paediatric patients [5, 26, 32]. A study of caregivers and adult psychiatry patients found an association between previous telehealth use and preference for future use, suggesting that those with previous telehealth experience had an increased preference for using telehealth in the future [5]. Our study found no association between previous use of telehealth (for general, or ADHD-specific, appointments) and willingness to use telehealth in the future, though an association was found with pre-pandemic experience and willingness to use telehealth. This may indicate that willingness does not differ based on experience gained, but based on when telehealth was initiated (before pandemic vs during pandemic). It is recognised that the pandemic was a time of psychological stress and burden for parents of children with neurodevelopmental conditions [33], which may contribute to unwillingness to use telehealth based on stressful experiences during the pandemic. Also, our study did not find an association between the amount of times telehealth was used and willingness to use telehealth. We compared those who used telehealth five times or less and those who used it six times or more, which may not have been the most suitable way to categorise inexperienced versus established users.

### Implications for practice and research

Parents expressed concerns about the quality of diagnostic assessments via telehealth, with parents largely interested in having in-person consultations for these appointments. There is still limited research on the effectiveness of telehealth for ADHD and neurodevelopmental assessments [34, 35], so this is an urgent area of future research. Furthermore, concerns about the perceived quality of telehealth may arise from parents’ lack of understanding of the process. It is worth noting that approximately half of the sample had no experience of telehealth for ADHD appointments. Patient satisfaction is a complex concept, particularly in ADHD care [36], where parents may have inaccurate expectations about what constitutes “good quality” care. Providers have a responsibility to explain this process to parents, including correcting misconceptions. Furthermore, there may be other unrelated factors, such as the parent’s opinion of the clinician, that impact parents’ perceptions of the “quality” of telehealth. Understanding these views on how parents conceptualise “good quality” care would highlight specific areas of concern and reluctance among parents. These areas could subsequently be addressed by the clinician, by information sessions, or through educational media campaigns. Furthermore, providing training to parents on the effectiveness of telehealth could help to increase their trust and confidence in the process.

Augmenting telehealth models through the addition of remote patient monitoring (e.g. heart rate, blood pressure) could also ease the concerns of parents who believed the “hands-on” element of in-person care was missing. The success of telehealth has been found among other mental health conditions which require careful physical monitoring, such as eating disorders [37]. In the present sample, caregivers reported high use of technological devices so incorporating use of home monitoring of heart rate and blood pressure could be a feasible option. Adapting telehealth to include physical monitoring could allow for safe, remote care, particularly given the high rates of physical comorbidities in ADHD and adverse effects of medications (e.g. cardiac adverse events) [38].

A direction for future research is surveying and interviewing children and adolescents themselves on their views of telehealth. Parents, and children and adolescents with ADHD, may have differing opinions of health services [39], so it is important to understand both views to provide patient-centred care. For example, it is acknowledged that adolescents may experience additional complexities using telehealth in terms of maintaining privacy and confidentiality [31, 40]. Another potential research direction would be to investigate differences in telehealth use and acceptability between younger children and older children/adolescents. The present survey did not allow for a comparison of this nature, but understanding differences in experiences and acceptability among children of various ages would allow telehealth to be adapted to suit the differing needs and preferences of younger children versus adolescents, and their parents. Furthermore, collecting data on co-occurring conditions (e.g. autism spectrum disorder, severe mental illness) and symptom severity may help to understand some of the challenges faced by children and adolescents with ADHD with regards to telehealth.

The timeliness and efficiency of telehealth services (i.e. reduced waiting times, seen quicker than in-person appointment) has also been mentioned as an advantage by parents in other studies [5, 26], though this has not been widely investigated in neurodevelopmental conditions [35]. In the context of post-pandemic waiting lists, this benefit is particularly valued among parents. One potential area of research, as discussed by other authors [5, 34], would be to investigate these potential service efficiencies and cost-savings though economic evaluations. In addition, understanding if these efficiency benefits are experienced by clinicians would be useful. Finally, participants were not asked if they accessed healthcare publicly or privately, so understanding views in the context of this information may be insightful.

### Strengths and limitations

This study has some limitations that should be considered. Firstly, whilst the study reached the minimum sample size required from the a apriori sample size calculation, it is important to acknowledge the low response rate of the survey. A low response rate is not optimal when making inferences from cross-sectional data, so caution must be taken when interpreting the findings. However, in the absence of any data, the survey provides valuable information about the views of caregivers of children and adolescents with ADHD on telehealth. Recruitment and engagement of caregivers in paediatric research is a challenge, so methods to improve response rates among this group, particularly male caregivers, should be researched [41]. The low response rate may limit also the representativeness of the survey; there was a higher proportion of female children and adolescents in this study than the proportion reported internationally [42].

With a self-selected sample there is a risk of response bias towards those who have strong opinions for, and against, telehealth. Another consideration is that the survey was distributed via email and through an ADHD organisation, so there is a risk of response bias towards those with access to email and internet and towards those prone to help-seeking. A final limitation is that we did not collect further demographic information, such as socio-economic status, so the impact of these characteristics on willingness to use telehealth could not be assessed. Whilst additional questions may have burdened participants, collecting this information would be useful, particularly when low socio-economic status has been associated with higher risk of ADHD diagnosis [43] and less accessibility to telehealth services [44].

The study has many strengths including addressing knowledge gaps and providing valuable insights into the views of parents and caregivers on the use of telehealth for ADHD. The survey was specifically designed from published literature and underwent input from ADHD Ireland and child and adolescent psychiatrists. Whilst the use of a study-specific survey was valuable to provide relevant and practical information, it was also necessary given the lack of a validated instrument to examine parents’ and caregivers’ perceptions of telehealth for ADHD. In particular, the question about caregivers’ ADHD diagnosis was not from a validated instrument. Future studies should endeavour to use validated measure of self-report for caregivers’ ADHD diagnosis, such as the Adult ADHD Self-Report Scale (ASRS) Screener [45]. Finally, the sample comprised parents and caregivers with, and without, experience with telehealth, meaning that the findings may be applicable to both groups of parents.

## Conclusions

This study explored parents’ and caregivers’ perceptions of telehealth for ADHD. We found that parents are willing to use telehealth for ADHD appointments, appreciating improvements to the family routine and the opportunity to access timelier care. However, parents have concerns about the quality of diagnostic assessments, which warrants further research and targeted approaches to address these concerns. Suggested areas for future research include exploring the views of children and adolescents with ADHD and continuing research on the effectiveness and cost-effectiveness of remote ADHD care. Taken together, the findings emphasise the importance of understanding and addressing the needs of children and adolescents with ADHD, and their families, for the successful use of telehealth.

## Supplementary Information

Below is the link to the electronic supplementary material.

Supplementary file1 (DOCX 265 KB)

## Data Availability

The datasets generated and/or analysed during the current study are not publicly available due to the protection of the privacy and confidentiality of the participants but are available from the corresponding author on reasonable request.

## Abbreviations

ADHD: Attention-deficit/hyperactivity disorder
CAMHS: Child and adolescent mental health services
SD: Standard deviation
UK: United Kingdom
