# Supplementary material for: The use of telehealth in attention-deficit/hyperactivity disorder: a survey of parents and caregivers
Source: Eur Child Adolesc Psychiatry. 2024 May 16;33(12):4247–57. doi: 10.1007/s00787-024-02466-y (PMC11618160; doi:10.1007/s00787-024-02466-y)
Supplement: Supplementary file 1 — Supplementary file1 (DOCX 265 KB) [file 787_2024_2466_MOESM1_ESM.docx]

**Supplementary material**

The use of telehealth in attention-deficit/hyperactivity disorder: a survey of parents and caregivers

Emer Galvin^1^, Blánaid Gavin, Ken Kilbride, Shane Desselle, Fiona McNicholas, Shane Cullinan, John Hayden

^1^ School of Pharmacy and Biomolecular Sciences, Royal College of Surgeons in Ireland, Dublin, Ireland.

Corresponding author: Emer Galvin, emergalvin20@rcsi.ie.

European Child & Adolescent Psychiatry

**Appendices**

Appendix 1. Research call

Appendix 2. Survey

Appendix 3. CHERRIES checklist

Appendix 4. Supplementary tables

Table S1. Satisfaction with telehealth

Table S2. Willingness to use telehealth

Appendix 5. Quotes from free-text comments

**Appendix 1. Research call**

The following research call was advertised on the ADHD Ireland website and distributed to their mailing list via email.


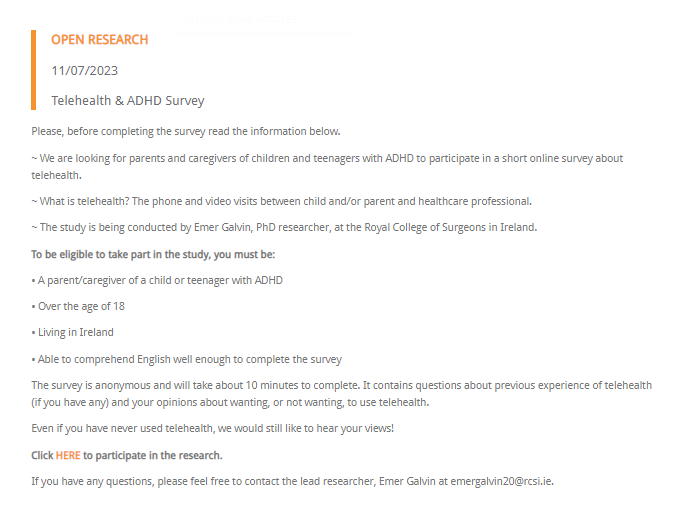


**Appendix 2. Survey**

**Telehealth & ADHD Survey**

This survey is about your views on using telehealth for your child's ADHD management and care. We define "telehealth" as video and/or phone visits between a healthcare professional and you and/or your child.

Many of you have used telehealth in the form of phone and video appointments with your child's health care professional(s). We are interested in learning about your opinions and experiences (if you have any) of telehealth as a parent/caregiver and how telehealth can be used in a way that takes into account you and your child's needs.

Even if you have not used telehealth before, we would still like to get your opinion.

Thank you very much for taking the time to complete this survey.

**Information Sheet**

You are being invited to take part in a research study as part of a PhD project at the Royal College of Surgeons in Ireland, Dublin. Our study is looking to explore parents' and caregivers' opinions of the use of telehealth for children and adolescents with attention-deficit/hyperactivity disorder (ADHD). "Telehealth" is a word used to describe phone and video visits between you and/or your child, and a healthcare professional. Even if you have no previous experience with telehealth, we are still interested in hearing your thoughts.

Before you decide whether or not you wish to take part, please read the information provided below carefully. It is important that you fully understand what the research is about. You should clearly understand the risks and benefits of taking part in this study so that you can make an informed decision. Please take the time to discuss your participation with friends and family. If you have any questions about the study, please do not hesitate to contact the Principal Investigator, Emer Galvin (emergalvin20@rcsi.ie).

You don't have to take part in the study and you can change your mind at any time up to the point of submitting the survey. Even if you have started the survey, you can still opt out, without giving us a reason. Thank you for your time and consideration.

**Why is this study being done?**

The COVID-19 pandemic caused a number of changes to how healthcare was delivered. One such change was that many face-to-face visits between patients and health care professionals changed to phone and/or video visits. We call these "telehealth visits". We are interested in the use of telehealth in the management and care of children and adolescents with ADHD.

This study is being done to explore parents' and caregivers' opinions and experiences (if you have any) of telehealth. Telehealth been used widely since the pandemic and it is important to understand people's experiences with these types of visits. In this study, we want to understand both the positive and negative aspects of telehealth. We want to understand people's views in order to inform recommendations of how telehealth can be used in the management and care of children and adolescents with ADHD.

**Who is organising the study?**

This study is being organised by Emer Galvin, who is a PhD student at the School of Pharmacy and Biomolecular Sciences at the Royal College of Surgeons in Ireland. She is doing this research as part of her PhD degree. Her research is interested in the use of telehealth in mental health services in the COVID-19 pandemic and beyond. Her PhD is funded by a Clement Archer Scholarship.

**Why am I being asked to take part?**

You have been asked to take part because you may have the perspectives we need. We would like to gather information from you and others to help us to understand more about parents' and caregivers' views of telehealth for their child with ADHD.

You are eligible to take part if:

You are a parent/caregiver of a child or adolescent with ADHD

You are over the age of 18

You live in Ireland

You can comprehend English well enough to complete this survey

How will the study be carried out? / What will happen to me if I agree to take part?

If you decide that you are happy to take part in the study, we will ask you to fill out a survey. The survey should take about 10 minutes to complete. The survey will ask questions about yourself and your child (e.g. age, gender, county of residence) to understand who is taking part in the survey. The survey will ask questions about previous experience of telehealth (if you have any) and your opinions about how telehealth can be used for ADHD management and care. The survey will also ask about reasons for wanting, or not wanting, to use telehealth.

After we have collected and analysed the information from all participants we will use it to help inform the use of telehealth for young people with ADHD and their families. We will also publish the findings in an academic journal and present them at academic conferences so that we can share our findings.

Your participation in this study is voluntary. Any information provided as part of the study will be encrypted and will follow data protection guidelines to ensure confidentiality. You participation will be anonymous and it will not be possible to identify you from your responses. If you provide potentially-identifiable information, this information will be removed to protect your identity.

You can withdraw from the study at any time up to the point of submitting the survey. Once the survey has been submitted it will no longer be possible to withdraw from the study. This is because your responses will be anonymous and it will not be possible to identify your responses from those of other participants.

**What are the possible benefits of taking part?**

Your co-operation with this study is very much appreciated as it will help to improve the use of telehealth for young people with ADHD and their families. Currently, there is not a lot of research on the use of telehealth for child and adolescent ADHD management and care. Our findings will help us provide information on how to improve the use of telehealth for young people with ADHD and their families.

**What are the possible risks of taking part?**

We do not foresee any risks or disadvantages for participating in this study. It is unlikely but possible that you may become uncomfortable while completing this survey. In the instance that you may become uncomfortable, please contact the Principal Investigator (Emer Galvin).

**Is the study confidential?**

The research team have put in place several steps to make sure the study is confidential. Your participation in the study will be anonymous and we will not collect any identifying information such as names or email addresses. Any information that might make you identifiable to others will be removed before the data is shared with the rest of the research team.

All your information will be encrypted, anonymised and stored in a dedicated, password-protected folder on the RCSI secure server. Access will be managed by the Principal Investigator (Emer Galvin). Any publications from this project will not identify you in any way.

Once any publications arising from this research have been accepted for publication, all data relating to the study will be deleted from the secure folders by a member of the research team. The period for which the data will be retained will not exceed 5 years.

A copy of the information sheet can be downloaded using the below link.

[Attachment: "Participant Information Leaflet.pdf"]

**Consent Form**

Please complete the following consent form and click on "Next Page" to begin the survey

1. I have read and understood the Information Sheet about this research project. The information has been explained to me in writing. I have been able to ask questions, which have been answered to my satisfaction (You are free to contact the lead researcher by phone or email).

2. I understand that I don't have to take part in this study and that I can opt before finishing the survey. I understand that I don't have to give a reason for opting out.

3. I am aware of the potential risks and benefits of this research study.

4. I have been assured that information about me will be kept private and confidential.

5. I consent to take part in this research study having been fully informed of the risks and benefits.

6. I give informed explicit consent to have my data processed as part of this research study.

**Demographic questions**

This section has some questions about yourself and your child to help us understand who is taking part in this survey.

1. What age are you?
   1. 18-24
   2. 25-34
   3. 35-44
   4. 45-54
   5. 55-64
   6. 65 and over
2. What is your gender?
   1. Male
   2. Female
   3. Other
      1. Please specify “Other”:
3. What is your relationship to the child?
   1. Parent
   2. Guardian
   3. Relative
   4. Other
      1. Please specify “Other”:
4. What county do you live in?
   1. Antrim
   2. Armagh
   3. Carlow
   4. Cavan
   5. Clare
   6. Cork
   7. Derry
   8. Donegal
   9. Down
   10. Dublin
   11. Fermanagh
   12. Galway
   13. Kerry
   14. Kildare
   15. Kilkenny
   16. Laois
   17. Leitrim
   18. Limerick
   19. Longford
   20. Louth
   21. Mayo
   22. Meath
   23. Monaghan
   24. Offaly
   25. Roscommon
   26. Sligo
   27. Tipperary
   28. Tyrone
   29. Waterford
   30. Westmeath
   31. Wexford
   32. Wicklow
5. Would you describe the place where you live as …
   1. a big city
   2. the suburbs or outskirts of a big city
   3. a small city or town
   4. a country village
   5. a farm or home in the country
   6. Other
      1. Please specify “Other”:
6. What is your employment status?
   1. Full-time
   2. Part-time
   3. Unemployed
   4. Retired
   5. Other
      1. Please specify “Other”:
7. Have you been diagnosed with ADHD yourself?
   1. Yes
   2. No
   3. No, but I think I might have ADHD
8. How many children with ADHD do you have?
   1. 1
   2. 2
   3. 3
   4. 4 or more

Child 1

1. What age is your child? ____
2. What is the gender of your child?
   1. Male
   2. Female
   3. Other

Child 2

1. What age is your child? ____
2. What is the gender of your child?
   1. Male
   2. Female
   3. Other

Child 3

1. What age is your child? ____
2. What is the gender of your child?
   1. Male
   2. Female
   3. Other

Child 4

1. What age is your child? ____
2. What is the gender of your child?
   1. Male
   2. Female
   3. Other

**Technology use**

1. Which of these devices do you have access to in your home? (Tick all that apply)
2. Smartphone
3. Mobile phone that is not a smartphone
4. Desktop computer
5. Laptop computer
6. Tablet
7. Gaming console
8. How often do you use the internet (across all devices)
9. Almost constantly
10. Several times a day
11. About once a day
12. Several times a week
13. Once a week
14. Less than once a week
15. Never
16. How often do you use video-based platforms (e.g. FaceTime, Zoom, WhatsApp video) to communicate with others?
17. Almost constantly
18. Several times a day
19. About once a day
20. Several times a week
21. Once a week
22. Less than once a week
23. Never

**Telehealth experience**

1. Have you used telehealth (phone and/or video visits) for health appointments **in general** (unrelated to your child’s ADHD)?
   1. Yes
   2. No
2. Have you used telehealth (phone and/or video visits) for appointments **regarding your child’s ADHD?**
   1. Yes
   2. No
3. (If Yes to Q2) How many times have you used telehealth (phone and/or video visits) for **regarding your child’s ADHD?**
   1. Once
   2. Five times or less
   3. More than 6 times
4. (If Yes to Q2) How were these telehealth visits conducted?
   1. Mainly over video
   2. Mainly over phone
   3. Equal mix of phone or video
5. (If Yes to Q2) What types of appointments have **you and/or your child** used telehealth (phone and/or video visits) for? Please tick all that apply.
   1. General appointment
   2. Check-in appointment
   3. Therapeutic/psychological intervention
   4. Diagnostic assessment
   5. Medication review
   6. Group intervention
   7. Other
      1. Please specify
6. Before the COVID-19 pandemic, had **you** used telehealth (phone and/or video visits) for any type of health appointment?
   1. Yes
   2. No

**Satisfaction with telehealth**

*(This section appeared if participants answered “Yes” to “Have you used telehealth (phone and/or video visits) for appointments regarding your child’s ADHD?”*)

These questions are about your experience with using telehealth (phone and/or video visits) for appointments regarding your child's ADHD.

1. Compared to in-person visits, the quality of telehealth visits were
   1. Much worse
   2. Somewhat worse
   3. Equally good
   4. Somewhat better
   5. Much better
2. How comfortable did you feel communicating with the healthcare professional via telehealth?
   1. Very uncomfortable
   2. Uncomfortable
   3. Neutral
   4. Comfortable
   5. Very comfortable
3. How comfortable did **your child** feel communicating with the healthcare professional via telehealth?
   1. Very uncomfortable
   2. Uncomfortable
   3. Neutral
   4. Comfortable
   5. Very comfortable
4. How easy was it to use the technology for telehealth visits?
   1. Very difficult
   2. Difficult
   3. Neutral
   4. Easy
   5. Very easy
5. How satisfied were you with the privacy/security of the telehealth visits?
   1. Very dissatisfied
   2. Somewhat dissatisfied
   3. Neither satisfied nor dissatisfied
   4. Somewhat satisfied
   5. Very satisfied
6. Overall how satisfied were you with telehealth (phone/video) visits?
   1. Very dissatisfied
   2. Somewhat dissatisfied
   3. Neither satisfied nor dissatisfied
   4. Somewhat satisfied
   5. Very satisfied

**Views on telehealth**

We would like to know your views on telehealth especially regarding its potential uses and reasons for wanting to use/not use telehealth

Even if you do not have experience with telehealth or know a lot about it, we would still like to capture your opinion.

1. Would you be willing to use telehealth for future appointments regarding your child’s ADHD?
   1. Yes
   2. No
2. What proportion of the following appointment types would you be willing to use telehealth for?


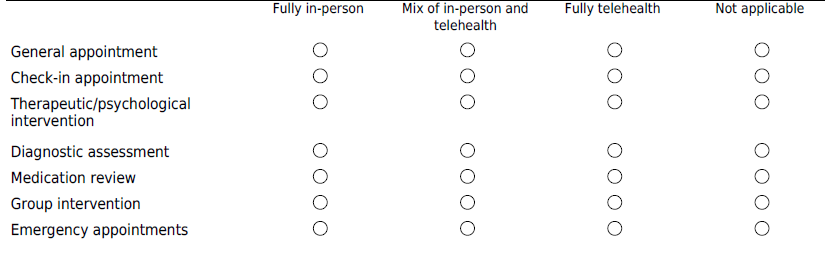


**Views on telehealth continued**

1. Why would you be willing to use telehealth for future appointments regarding your child's ADHD?

Please rank up to five reasons, from most important to you (1st) to least important (5th).

If this question is not relevant to you, please leave it blank.


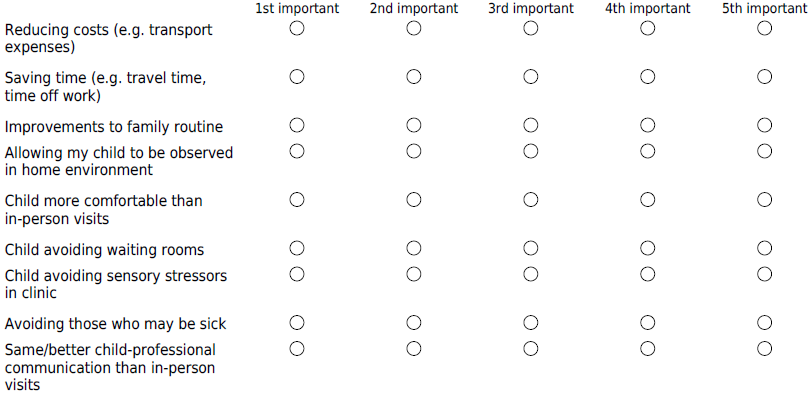


1. Are there any other reasons why you would be willing to use telehealth visits? Please explain. (Open-ended)

**Views on telehealth continued**

1. Why would you not be willing to use telehealth for future appointments regarding your child's ADHD?

Please rank up to five reasons, from most important to you (1st) to least important (5th).

If this question is not relevant to you, please leave it blank.


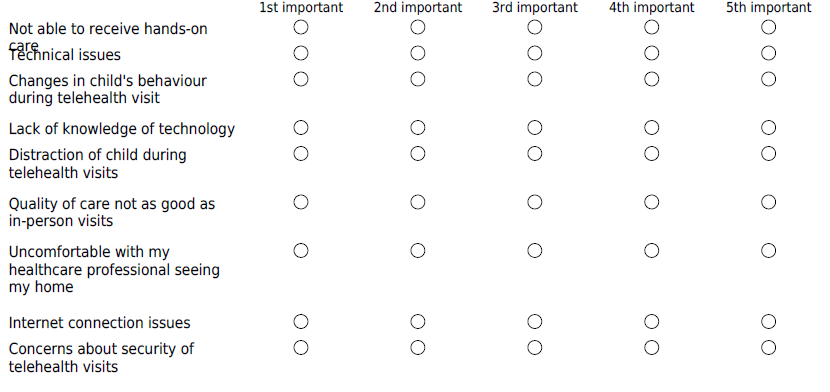


1. Are there any other reasons why you would not be willing to use telehealth visits? Please explain. (Open-ended)

**Additional comments**

Is there anything else you would like to let us know about your opinions or experience of telehealth? (Open–ended)

- End of survey -

**Appendix 3. Checklist for Reporting Results of Internet E-Surveys (CHERRIES)**

| ***Checklist Item*** | ***Explanation*** | ***Description*** |
| --- | --- | --- |
| Describe survey design | Describe target population, sample frame. Is the sample a convenience sample? (In “open” surveys this is most likely.) | Parents and caregivers of children and adolescents with attention-deficit/hyperactivity disorder. |
| IRB approval | Mention whether the study has been approved by an IRB. | The study has been approved by the Royal College of Surgeons in Ireland Research Ethics Committee (Approval number: 212627453). |
| Informed consent | Describe the informed consent process. Where were the participants told the length of time of the survey, which data were stored and where and for how long, who the investigator was, and the purpose of the study? | Informed consent was obtained by all participants before the commencement of the survey. The participants were informed of the length of the survey, which data was stored, where and for how long the data would be stored for, who the principal investigator was, and the purpose of the study. This information was presented to the participants in an information sheet before the commencement of the survey. |
| Data protection | If any personal information was collected or stored, describe what mechanisms were used to protect unauthorized access. | No personal information was collected or stored, to protect the identities of the participants. No geo-location data or IP addresses were collected. Study data was stored in a password-protected REDCap account, which only the lead investigator/author had access to (EG). Data was downloaded on to a password-protected folder on the RCSI secure server. |
| Development and testing | State how the survey was developed, including whether the usability and technical functionality of the electronic questionnaire had been tested before fielding the questionnaire. | The survey was developed from a literature review and input from child and adolescent psychiatrists, in addition to a member of ADHD Ireland. The survey went under usability and functionality testing with lay members of the public. The survey underwent further pilot-testing with parents/caregivers of children with ADHD, to ensure readability. |
| Open survey versus closed survey | An “open survey” is a survey open for each visitor of a site, while a closed survey is only open to a sample which the investigator knows (password-protected survey). | The survey was an open survey. |
| Contact mode | Indicate whether or not the initial contact with the potential participants was made on the Internet. (Investigators may also send out questionnaires by mail and allow for Web-based data entry.) | The survey was distributed by email to potential participants and advertised on the ADHD Ireland website. |
| Advertising the survey | How/where was the survey announced or advertised? Some examples are offline media (newspapers), or online (mailing lists – If yes, which ones?) or banner ads (Where were these banner ads posted and what did they look like?). It is important to know the wording of the announcement as it will heavily influence who chooses to participate. Ideally the survey announcement should be published as an appendix. | The survey was posted on the ADHD Ireland website on their “Research Calls” section. This research call can be seen in Appendix 1.  The survey advertisement was also shared on Twitter with the following announcement “Together with @RCSI_Irl we would like to invite parents of caregivers of ADHD children and teens to participate in the new research about the phone and video visits between child and/or parent and healthcare professional. click here > link” |
| Web/E-mail | State the type of e-survey (eg, one posted on a Web site, or one sent out through e-mail). If it is an e-mail survey, were the responses entered manually into a database, or was there an automatic method for capturing responses? | The survey was distributed via email and also posted on the ADHD Ireland website. The survey responses were automatically captured using REDCap software. |
| Context | Describe the Web site (for mailing list/newsgroup) in which the survey was posted. What is the Web site about, who is visiting it, what are visitors normally looking for? Discuss to what degree the content of the Web site could pre-select the sample or influence the results. For example, a survey about vaccination on a anti-immunization Web site will have different results from a Web survey conducted on a government Web site | The survey was posted on www.adhdireland.ie. The survey was also distributed by ADHD Ireland on their weekly mailing list. This website provides up-to-date information, resources and networking opportunities to individuals with ADHD, parents of children with ADHD and the professionals who serve them. |
| Mandatory/voluntary | Was it a mandatory survey to be filled in by every visitor who wanted to enter the Web site, or was it a voluntary survey? | This was a voluntary survey. |
| Incentives | Were any incentives offered (eg, monetary, prizes, or non-monetary incentives such as an offer to provide the survey results)? | No incentives were offered to participants. |
| Time/Date | In what timeframe were the data collected? | Data were collected in July and August 2023. |
| Randomization of items or questionnaires | To prevent biases items can be randomized or alternated. | Survey items were not randomized or alternated. |
| Adaptive questioning | Use adaptive questioning (certain items, or only conditionally displayed based on responses to other items) to reduce number and complexity of the questions. | Adaptive questioning was used. Some items were conditionally displayed based on responses to other items.  For example, questions about previous use of telehealth were only displayed if participants indicated that they had previous experience with telehealth. |
| Number of Items | What was the number of questionnaire items per page? The number of items is an important factor for the completion rate. | 1^st^ page: Information leaflet & consent form  2^nd^ page: 10 questions  3^rd^ page: 3 questions  4^th^ page: 3 questions (plus 3 conditional questions)  5^th^ page: 6 conditional questions  6^th^ page: 2 questions  7^th^ page: 2 questions  8^th^ page: 2 questions  9^th^ page: 1 question |
| Number of screens (pages) | Over how many pages was the questionnaire distributed? The number of items is an important factor for the completion rate. | The survey was distributed over 9 pages. |
| Completeness check | It is technically possible to do consistency or completeness checks before the questionnaire is submitted. Was this done, and if “yes”, how (usually JAVAScript)? An alternative is to check for completeness after the questionnaire has been submitted (and highlight mandatory items). If this has been done, it should be reported. All items should provide a non-response option such as “not applicable” or “rather not say”, and selection of one response option should be enforced. | We did not enforce mandatory responses.  Some items included a “not applicable” response option. |
| Review step | State whether respondents were able to review and change their answers (eg, through a Back button or a Review step which displays a summary of the responses and asks the respondents if they are correct). | Respondents were able to review and change their answers through the use of a “Back button”. |
| Unique site visitor | If you provide view rates or participation rates, you need to define how you determined a unique visitor. There are different techniques available, based on IP addresses or cookies or both. | N/A |
| View rate (Ratio of unique survey visitors/unique site visitors) | Requires counting unique visitors to the first page of the survey, divided by the number of unique site visitors (not page views!). It is not unusual to have view rates of less than 0.1 % if the survey is voluntary. | N/A |
| Participation rate (Ratio of unique visitors who agreed to participate/unique first survey page visitors) | Count the unique number of people who filled in the first survey page (or agreed to participate, for example by checking a checkbox), divided by visitors who visit the first page of the survey (or the informed consents page, if present). This can also be called “recruitment” rate. | N/A |
| Completion rate (Ratio of users who finished the survey/users who agreed to participate) | The number of people submitting the last questionnaire page, divided by the number of people who agreed to participate (or submitted the first survey page). This is only relevant if there is a separate “informed consent” page or if the survey goes over several pages. This is a measure for attrition. Note that “completion” can involve leaving questionnaire items blank. This is not a measure for how completely questionnaires were filled in. (If you need a measure for this, use the word “completeness rate”.) | 100/121 = 82.6% |
| Cookies used | Indicate whether cookies were used to assign a unique user identifier to each client computer. If so, mention the page on which the cookie was set and read, and how long the cookie was valid. Were duplicate entries avoided by preventing users access to the survey twice; or were duplicate database entries having the same user ID eliminated before analysis? In the latter case, which entries were kept for analysis (eg, the first entry or the most recent)? | We did not collect data on cookies. |
| IP check | Indicate whether the IP address of the client computer was used to identify potential duplicate entries from the same user. If so, mention the period of time for which no two entries from the same IP address were allowed (eg, 24 hours). Were duplicate entries avoided by preventing users with the same IP address access to the survey twice; or were duplicate database entries having the same IP address within a given period of time eliminated before analysis? If the latter, which entries were kept for analysis (eg, the first entry or the most recent)? | We did not collect data on IP addresses. |
| Log file analysis | Indicate whether other techniques to analyze the log file for identification of multiple entries were used. If so, please describe. | We did not conduct a log file analysis. |
| Registration | In “closed” (non-open) surveys, users need to login first and it is easier to prevent duplicate entries from the same user. Describe how this was done. For example, was the survey never displayed a second time once the user had filled it in, or was the username stored together with the survey results and later eliminated? If the latter, which entries were kept for analysis (eg, the first entry or the most recent)? | This was an open survey. |
| Handling of incomplete questionnaires | Were only completed questionnaires analyzed? Were questionnaires which terminated early (where, for example, users did not go through all questionnaire pages) also analyzed? | We analysed incomplete and completed surveys. |
| Questionnaires submitted with an atypical timestamp | Some investigators may measure the time people needed to fill in a questionnaire and exclude questionnaires that were submitted too soon. Specify the timeframe that was used as a cut-off point, and describe how this point was determined. | We did not use a timeframe as a cut-off point. |
| Statistical correction | Indicate whether any methods such as weighting of items or propensity scores have been used to adjust for the non-representative sample; if so, please describe the methods. | We did not use weighting of items or propensity scores to adjust for non-representative sample. |

This checklist has been modified from Eysenbach G. Improving the quality of Web surveys: the Checklist for Reporting Results of Internet E-Surveys (CHERRIES). J Med Internet Res. 2004 Sep 29;6(3):e34 [erratum in J Med Internet Res. 2012; 14(1): e8.]. Article available at [https://www.jmir.org/2004/3/e34](https://www.jmir.org/2004/3/e34/)/; erratum available <https://www.jmir.org/2012/1/e8/>. Copyright ©Gunther Eysenbach. Originally published in the [Journal of Medical Internet](http://www.jmir.org) Research, 29.9.2004 and 04.01.2012.

This is an open-access article distributed under the terms of the Creative Commons Attribution License (<https://creativecommons.org/licenses/by/2.0/>), which permits unrestricted use, distribution, and reproduction in any medium, provided the original work, first published in the Journal of Medical Internet Research, is properly cited.

**Appendix 4. Supplementary tables**

**Table S1. Satisfaction with telehealth (n = 61)**

| **Statement** | **n (%)** |
| --- | --- |
| ***Quality of telehealth vs. in-person visits*** | |
| Much worse | 7 (11.5%) |
| Somewhat worse | 24 (39.3%) |
| Equally good | 27 (44.3%) |
| Somewhat better | 2 (3.3%) |
| Much better | 1 (1.6%) |
| ***Comfort in communicating with healthcare professional via telehealth*** | |
| Very uncomfortable | 4 (6.6%) |
| Uncomfortable | 7 (11.5%) |
| Neutral | 12 (19.7%) |
| Comfortable | 23 (37.7%) |
| Very comfortable | 15 (24.6%) |
| ***Comfort of child in communicating with healthcare professional via telehealth*** | |
| Very uncomfortable | 10 (16.4%) |
| Uncomfortable | 11 (18%) |
| Neutral | 12 (19.7%) |
| Comfortable | 10 (16.4%) |
| Very comfortable | 11 (18%) |
| Not applicable/missing | 7 (11.5%) |
| ***Ease of use of technology*** | |
| Very difficult | 0 (0%) |
| Difficult | 6 (9.8%) |
| Neutral | 12 (19.7%) |
| Easy | 26 (42.6%) |
| Very easy | 17 (27.9%) |
| ***Satisfaction with privacy/security of telehealth visit*** | |
| Very dissatisfied | 3 (4.9%) |
| Somewhat dissatisfied | 2 (3.3%) |
| Neither satisfied or dissatisfied | 13 (21.3%) |
| Somewhat satisfied | 15 (24.6%) |
| Very satisfied | 28 (45.9%) |
| ***Overall satisfaction with telehealth*** | |
| Very dissatisfied | 4 (6.6%) |
| Somewhat dissatisfied | 11 (18%) |
| Neither satisfied or dissatisfied | 9 (14.8%) |
| Somewhat satisfied | 19 (31.2%) |
| Very satisfied | 17 (27.9%) |
| Missing | 1 (1.6%) |

**Table S2. Willingness to use telehealth**

|  | **Willingness to use telehealth in the future** | | |  |
| --- | --- | --- | --- | --- |
| **Variable** | **Yes** | **No** | **Total** | **Overall**  **p-value*** |
| Age (n = 110)  *44 and under*  *45 and above* | 27 (24.6%)  64 (58.2%) | 4 (3.6%)  15 (13.6%) | 31 (28.2%)  79 (71.8%) | .58^b^ |
| Gender (n = 107)  *Male*  *Female* | 11 (10.3%)  79 (73.8%) | 1 (0.9%)  16 (15%) | 12 (11.2%)  95 (88.8%) | .69^b^ |
| Previously used for general appointment (n = 110)  *Yes*  *No* | 53 (48.2%)  38 (34.6%) | 7 (6.4%)  12 (10.9%) | 60 (54.5%)  50 (45.5%) | .09^a^ |
| Previously used for ADHD appointment (n = 110)  *Yes*  *No* | 48 (43.6%)  43 (39.1%) | 12 (10.9%)  7 (6.4%) | 60 (54.5%)  50 (45.5%) | .41^a^ |
| Previously used before pandemic (n = 110)  *Yes*  *No* | 17 (15.5%)  74 (67.3%) | 0(0%)  19 (17.3%) | 17 (15.5%)  93 (84.6%) | .04^b^ |
| Times used (n = 60)  *Five times or less*  *Six times or more* | 28 (46.7%)  20 (33.3%) | 7 (11.7%)  5 (8.3%) | 35 (58.3%)  25 (41.7%) | .99^a^ |
| Satisfaction (n = 59)  *Dissatisfied*  *Satisfied/Neutral* | 6 (10.2%)  41 (69.5%) | 8 (13.6%)  4 (6.8%) | 14 (23.7%)  45 (76.3%) | < .001^b^ |
| Quality of care, (n = 60)  *Worse*  *Equal/Better* | 19 (31.7%)  29 (48.3%) | 11 (18.3%)  1 (1.7%) | 30 (50%)  30 (50%) | .002^b^ |

*For categorical variables a chi-squared test or Fishers exact test (when expected cells <5) was used

^a^ Chi-squared test

^b^ Fisher’s exact test

**Appendix 5. Quotes from free-text comments**

**Reasons for wanting to use telehealth in the free-text comments**

| **Reason** | **Illustrative quotes** |
| --- | --- |
| Quicker access and increased availability of appointments via telehealth | *“It should reduce waiting lists for all of services were run more efficiently and it helps to do that”*  *“To get quicker access to clinicians (including psychologists, psychiatrists, social workers, occupational therapists, etc.).”*  *“Telehealth would open up availability for appointments and could allow for a wider variety of providers and types of services.”*  *“Overall it's a good option especially for Kerry & remote areas where we do not have the experience at the moment in the senior doctors”* |
| Telehealth useful for check-in and medication appointments | *“parents are capable of relaying how a medication trial is going over the phone and should be allowed to do so as otherwise you're weeks waiting for an in person appointment while a child may have issues with the medication they need resolved! Phone check ins should be allowed as it supports best practice titration, after two weeks ask the parents how is it going/side effects etc. Instead you're just left to it for weeks.”*  *“It remains a very pragmatic complement to in-person care.”*  *“I think some visits like assessment for diagnosis are better done in person but other visits would be more efficient over the phone or video calls”*  *“I am happy to do telehealth calls for medication reviews.”* |
| View that child is more comfortable at home than at in-person appointments | *“Admittedly it is challenging to get her to go to a doctors, she's not comfortable there either.”*  *“Prolongation of allowing my child to be observed in home environment. Child 2 who had evited any constructive relation with psychology, succeeded in surmounting his difficulties by creating a relation of confidence from the comfort of his own home during COVID, which has been maintained since in her office.”*  *“less anxiety around the social aspects of these visits for my daughter. more flexibility around where we are during the visits e.g., we can move to a different room/ sit in the car depending on how her other sibling is. “*  *“Child more comfortable in home setting.”* |
| Convenience: to save time, taking time off work, and getting childcare | *“My sons' ADHD and ASD assessments and diagnoses were all performed by video appointment. Doing them remotely made things much easier for us as we would have had to travel to Cork for all of our appointments. This would have entailed the bones of a 4 hour round trip, plus having to take a day's annual leave. I was lucky enough to be able to work from home on the day of the appointments, and could just clock out for the duration of the calls. In my opinion telehealth was one of the good things that Covid brought to the world (the other being mRNA vaccines)”*  *“less time out of school for appointments. “*  *“The clinic hours are incompatible with work and school. Being able to log in from these locations avoids me having to take annual leave that could be spent with family relaxing and my boys missing minimal school hours ie 30 minutes vrs the travel, park, waiting, delays, over stimulated and inevitably not making it back into class”*  *“it's easier for us as a family I have 3 kids on the spectrum,travelling is so hard for us with sensory issues and cost also the consequences after travelling with the kids.”*  *“they gave us six sessions with a senior psychologist online, which both parents could attend as it is so much easier via telehealth, rather than travelling a half hour away mid-day when we both worked in Dublin city centre. Cannot actually express how much the online/phone option helped us pre-remote working plus oppositional tween who resisted every single appointment.”* |
| To avoid stress and disruption of attending in-person appointments | *“I have ADHD and sometimes I feel uncomfortable in my own body. Also, getting ready and getting somewhere on time takes a lot of steps, organising and it's really stressful. It seems so unnecessary to be in person for some appointments. So a mix of being able to stay at home and not feeling self conscious about my body and not having to stress myself trying to get to an appointment on time is really helpful. Having to get to appointments can really effect my health, sometimes when I have to go to appointments in person I experience chronic fatigue afterwords so that makes my ADHD symptoms worse.”*  *“I am autistic and find it less intimidating and stressful to meet over zoom. It also avoids the stress of getting my son organised to attend an appointment - which can be quite challenging. “*  *“reduces parent child conflict with time management. Child can get annoyed when in person appointments run late.”*  *“a benefit of telehealth is there are less transitions for the child to complete to attend an appointment. there is also less pressure on the parent to be at a clinic for an appointment. I also find that after a telehealth appointment (my own or my childs), we are less burnt out then if it was in clinic. this means I've more of a chance getting her to school after an early morning telehealth appointment. if its clinic based, I try to make it afternoon based as she will not go back afterwards.”* |
| In emergency situations or if child was ill | *“emergency appointment and unable to travel”*  *“Main reason now is Speed of Response (more than Saving Time in list above) i.e. crisis occurs and make in-person appointment but it may take a week or two to see professional and crisis has passed and hard to explain at this remove.”*  *“Emergencies”*  *“only if they had something contagious, e.g. chickenpox or Covid”* |
| Suited family situation | *“Easier to get both of us parents there at same time.”*  *“I will add from personal experience and support groups, this mostly helps women, there is a gendered element to the stress reduction aspect of health services changes. Men do not attend many appointments I've seen it in CAMHS for years. Online and phoned allowed my husband to be more involved, or both of us to be engaged on the treatment.”*  *“more than on parent could attend some of it”* |

**Reasons for not wanting to use telehealth in the free-text comments**

| **Reason** | **Illustrative quotes** |
| --- | --- |
| View that child experienced difficulty with, and distraction during, telehealth visits | *“My child hates video calls and would not interact with the team well on video My child hates group therapy on video even more! She would not engage in webinars or group sessions online but would attend face to face groups”*  *“I would not be willing to access telehealth for my child with ADHD. My child finds it difficult to access screens because of his neuro divergence. The consulting doctor would get more info from a physical in person visit than a phone call too.”*  *“My child becomes very distracted and hyper on video calls, and it's difficult for the clinician to properly question/assess him because of this.”*  *“I find my child is distracted by home environment and does not focus on doctor on screen. He is always keen to hit the red end button to finish consultations.”* |
| Preference and belief about necessity of in-person appointments | *“I would rather in-person interactions when it comes to my child however if I can have access and support from therapists or medics over the phone is would be very convenient for me and would save time.”*  *“Although we prefer telehealth there are appointments where you need in person care.”*  *“In reality my preference is always an in person visit , other than reviewing medication. However I would prefer televisit than no visit or longer time in between appointments”*  *“I'm not sure video calls versus in person visits would be as effective. Human contact cannot be underestimated.”* |
| Belief that quality of interaction is impaired during telehealth viists | *“Missing a personal connection, not able to ask the questions that come up more organically in a 'live' setting, very artificial”*  *“Prefer in person to be honest, better flow of information exchange and engagement”*  *“I think a lot of non-verbal communication is lost when meetings are held via Zoom/Teams. I think a lot of nuance is lost. I think there is a convenience factor but the quality of the interaction is impaired.”* |
| Concerns about the accuracy of diagnoses and assessments over telehealth | *“I Don t honestly think it's possible to diagnose a person with ADHD or other via a zoom link. At the very least it should be face to face for a diagnosis and then maybe zoom with options to have a 6 monthly meeting in person.”*  *“Quick check in or review but I feel there are benefits lost from doing in person visits - correct assessment,”*  *“As noted, telehealth would have benefits for me in relation to supporting me to care for my child. But when it comes to assessing my child I would rather he interacted with professionals in-person. It would be very difficult for a healthcare professional to assess my childs ADHD over the phone or on video.”*  *“I would not be willing to access telehealth for my child with ADHD. My child finds it difficult to access screens because of his neuro divergence. The consulting doctor would get more info from a physical in person visit than a phone call too.”* |
| Inability of clinician to perform physical examinations | *“We've had medication reviews online where I still have to go and get BP taken elsewhere, or where BP and other observations were not completed. This is not good enough.”*  *“If a physical exam was needed”*  *“His psychiatrist has always tried her best to accommodate us and it has worked quite well but obviously she can't check my son's blood pressure etc”* |
